# Antioxidant Potential of *Fagonia arabica* against the Chemical Ischemia-Induced in PC12 Cells

**Published:** 2012

**Authors:** Ravindra Satpute, Rahul Bhattacharya, Rajpal S Kashyap, Hemant J Purohit, Jayant Y Deopujari, Girdhar M Taori, Hatim F. Daginawala

**Affiliations:** a *Toxicology Laboratory, Defence Research and Development Establishment, Mahanagar Palika Marg, Nagpur 440001, India.*; b *Division of Experimental Therapeutics, Defence Research and Development Establishment, Jhansi Road, Gwalior-474002, India.*; c *Biochemistry Research Laboratory, Central India Institute of Medical Sciences, Bajaj Nagar, Nagpur 440010, India. *; d *Environmental Genomics Unit, National Environmental Engineering Research Institute, Nehru Marg, Nagpur 440020, India.*

**Keywords:** Chemical ischemia, Energy metabolism, Cytotoxicity, PC 12 cells, *Fagonia arabica*

## Abstract

The imbalance between pro-oxidants and anti-oxidants leads to generation of oxygen/nitrogen free radicals which are implicated in several neurodegenerative diseases. *Fagonia arabica *is an ethno-pharmacologically important Ayurvedic herb known to have many medicinal properties like anti-inflammatory, analgesic and antipyretic effects. However, its antioxidant potential has not been investigated so far. The present study was designed to investigate the antioxidant potential of *F. arabica *and its neuroprotective effect on chemical ischemia induced in PC12 cells. Chemical ischemia was induced through exposing the cells to uncoupler of oxidative phosphorylation sodium azide (5.0 mM) and competitive inhibitor of glycolysis 2-deoxy-glucose (2.0 mM) for 2 h followed by 24 h reperfusion with normal culture medium. Total polyphenolic content (TPC) and antioxidant potential of the herb was measured using DPPH and ABTS•+ scavenging and ferric ion reducing antioxidant potential (FRAP) assays; its effect on neuroprotection and energy metabolism was also studied. The ischemic injury was characterized by impaired energy status as indicated by decreased ATP levels in the cells, accompanied by increased lactic acid content. Both the changes favourably responded to *F. arabica *and offered considerable neuroprotection from ischemia and helped to maintain the cellular viability and mitochondrial integrity of the cells. *F. arabica *showed considerable amount of TPC and antioxidant activity. This study reveals the antioxidant potential of *F. arabica *and its protective efficacy against ischemia/reperfusion mediated cell death. *F. arabica *thus can be considered for further studies for the development of the prophylactic or therapeutic agent for the treatment of ischemic stroke.

## Introduction

Ischemic stroke is the third leading cause of death worldwide after coronary heart diseases and cancer (1, 2). With an increasing population of elderly people, stroke is becoming a major health issue worldwide. Cerebral ischemia is most often brought about by interruption of the blood flow to the brain producing hypoxia and hypoglycemia resulting in neuronal cell death (3). Since neurons are highly vulnerable to hypoxia and hypoglycemia, they sustain irreversible damage even if supply of oxygen and glucose is blocked for a short time. Cerebral ischemia triggers a complex series of biochemical and molecular mechanisms which impairs the neurologic functions through breakdown of cellular integrates which is mediated by excitotoxic glutamatergic signalling, ionic imbalance and reactive oxygen and nitrogen species (ROS and RNS) generation (4, 5). Although the duration of ischemia is an important determinant of subsequent damage, reperfusion does also play a prominent role in the neuronal damage (5). A sudden supply of oxygen and glucose to starved tissues results in the production of ROS and RNS that further potentiates the cell death.

One of the myriad of events following cerebral ischemia is oxidative stress which poses a threat to the brain environment (6). The brain has a number of characteristics which make it especially susceptible to free radical injury. Brain lipids are highly enriched in polyunsaturated fatty acids (PUFA), and many regions of the brain viz. substantia nigra and striatum have high concentrations of iron. Both PUFA and iron increase the susceptibility of brain cell membranes to lipid peroxidation (7). ROS has been implicated in the pathophysiology of many neurological disorders and brain dysfunctions. Cellular functions are critically altered with increased production of free radicals after brain injury, which are generated through different cellular pathways (8). Increased vulnerability of brain to oxidative damage leads to the impairment of brain antioxidant systems after ischemia/reperfusion, which is responsible for failure of cerebral energy metabolism (9). At the present state of knowledge, treatment of ischemic brain injury is far from adequate (10).

In Ayurveda, the Indian traditional health care system, many herbal formulations have been used as a neuroprotective agent (11-13). Since long, many herbal formulations have been in use for various ailments like hematological and hepatic disorders, and inflammatory conditions (14, 15). *Fagonia arabica *is a tropical herb belonging to family Zygophyllaceae, found in the entire Indian subcontinent and is commonly known as ‘Dhamasa’. It is a green shrub of 1 to 3 feet height found on calcareous rocks distributed throughout the Mediterranean region of Africa, Afghanistan, India and Pakistan (16). Different parts of this herb have been used to cure various ailments, namely hematological, neurological, endocrinological and inflammatory disorders (11-13, 17-19). It has also been reported to contain wide variety of antioxidants and triterpenoids saponins (20, 21). Its infusion is effective as a cooling agent in stomatitis. It is known to purify blood and also acts as a deobstruent (22). It is also used for skin diseases, small pox and for endothermic reaction in the body (23). The twigs of the plant are used as remedy for snake bite and also applied externally as paste on tumours and for the swellings of neck (16, 17, 22).

The objectives of this investigation were to determine the total polyphenol contents and characterize the free radical scavenging, ferric ion reducing capabilities of *F. arabica*. The neuroprotective activity of *F. arabica *and its effect on the cellular energy status have also been investigated in ischemia induced in neuron-like rat pheochromocytoma (PC12) cells.

## Experimental

*Chemicals*

All the chemicals used were of analytical grade. Sodium azide (NaN3), 2-deoxyglucose (2-DG), gallic acid, potassium peroxodisulfate, 2,2’-azinobis-(3-ethyl-benzothiazoline-6-sulfonic acid (ABTS), 1,1-diphenyl-2-picrylhydrazyl free radical (DPPH), 2,4,6-tri(2-pyridyl)-s-triazine (TPTZ), nerve growth factor (NGF), 3-(4,5-dimethylthiazol-2-yl)-2,5-diphenyl tetrazolium bromide (MTT), trypan blue, Dulbecco’s phosphate buffer saline (DPBS) and other chemicals of highest purity, and cell culture media, serum and buffer constituents were purchased from Sigma-Aldrich Co. (St. Louis, MO, USA). 6-hydroxy-2,5,7,8-tetramethylchromane-2-carboxylic acid (Trolox) was purchased from Acros Organics (New Jersy, USA). Anhydrous sodium carbonate, ferric chloride hexahydrate (FeCl3•6H2O), Folin-Ciocalteu phenol reagent, hydrochloric acid, glacial acetic acid, methanol and sodium acetate trihydrate were purchased from Merck (Darmstadt, Germany).

*Composition of calcium krebs ringer (CaKR) buffer*

The CaKR buffer (pH 7.4) used for ischemic cells was devoid of glucose while the one used for control cells contained 6 mM glucose. The modified CaKR buffer (without glucose) composed of 125 mM NaCl, 5 mM KCl, 2.5 mM HEPES-NaOH, 5 mM NaHCO3, 1.2 mM MgSO4, 1.2 mM KH2PO4, 1 mM CaCl2 mM, supplemented with 5 mM sodium azide and 2 mM 2-DG. 

*Plant material*

*F. arabica *was purchased from Innocon Foods Pvt. Ltd., Pune, India (Batch number: C/6473). Multiple solvents (methanol: isopropyl alcohol: acetone) were used by the manufacturer for the preparation of the extract. As described, the sample (herbal extract) was authenticated for their correct botanical identity. 100 mg of the brownish colored thick gel of the extract was dissolved in 10 mL of CaKR buffer and the suspension was shaken vigorously on a vortex mixer. The suspension was kept overnight at 4°C and decanted to remove the soluble supernatant, which was filtered through a 0.22 μ syringe filter. The filtrate was collected and used as a stock (10 mg/mL) for further experimentation.

*Cell culture, differentiation and determination of cell viability*

Rat pheochromocytoma cells (PC12) were purchased from NCCS Pune, India, and maintained in F-12 HAM, Kaighn’s modification (HAM’s F12K) medium supplemented with 15% donor’s horse serum, 3% fetal calf serum (FCS) and 1% antibiotic antimicotic solution (Himedia, India), containing 10,000 units Penicillin, 10 mg streptomycin and 25 μg Amphotericin B/mL of culture medium. The cells were routinely incubated in 50 mL, poly-L-lysine (molecular weight > 70,000 D) coated, tissue culture flasks in humidified atmosphere of 5% CO2 and 95% air at 37°C. For experimentation 2x104 and 6x104 cells were seeded in 24 well tissue culture plates (lysine coated) for ATP and MTT respectively, while 2 × 106 cells were used for all other assays. 50 ng/mL NGF was added to the well containing differentiation media and kept for 4 days to differentiate the PC12 cells from synaptic like neurons. Differentiation media was changed on every second day. Cell viability was determined through trypan blue dye exclusion and > 95% cell viability was considered optimum for each treatment.

*Induction of chemical ischemia and treatment of herbal extract*

Sodium azide (5.0 mM) and 2-DG (2.0 mM) were used to induce chemical hypoxia and hypoglycemia, respectively. The chemical ischemia was induced in PC12 cells by both metabolic inhibitors according to procedures described previously (24, 25). Once the monolayer was formed, the incubation media was removed and ischemic medium (CaKR buffer containing sodium azide and 2-DG) was introduced to cells for 2 h. Thereafter, this ischemic medium was removed and cells were washed twice with normal CaKR buffer followed by the addition of normal culture medium for 24 h, in the absence or presence of 10, 50 or 100 μg/0.2 mL concentration of herbal extract.

*Determination of total polyphenol content (TPC)*

*The TPC was measured using the method des*cribed by Wong *et al*., (26). An aliquot of 100 μL of cell extract was mixed with 2.5 mL of Folin-Ciocalteu phenol reagent (10X dilutions) and allowed to react for 5 min. Then 2.5 mL of saturated Na2CO3 solution was added and allowed to stand for 1 hr and absorbance of the reaction mixture was measured at 725 nm on Helios α spectrophotometer (Thermo Electron Corpn., USA) and TPC of the extract was expressed as mg gallic acid equivalents/ g dry weight of plant. 

*Determination of antioxidant activity*

*DPPH free radicals scavenging assay*

The DPPH free radical scavenging activity of each sample was determined colorimetrically, according to the method described by Leong and Shui (27). Briefly, a 0.1 mM solution of DPPH in methanol was prepared. The initial absorbance of the DPPH in methanol was measured at 515 nm and did not change throughout the period of assay. An aliquot (40 μL) of an extract (with appropriate dilution, if necessary) was added to 3 mL of methanolic DPPH solution. The change in absorbance at 515 nm was measured after 30 min. The antioxidant capacity based on the DPPH free radical scavenging ability of the extract was expressed as μM Trolox equivalents/ g dry weight of plant.

*Ferric reducing antioxidant potential (FRAP) assay*

The ability to reduce ferric ions was measured using a modified version of the method described by Benzie and Strain (28). An aliquot (200 μL) of an extract (with appropriate dilution, if necessary) was added to 3 mL of FRAP reagent (10 parts of 300 mM sodium acetate buffer at pH 3.6, 1 part of 10 mM TPTZ solution and 1 part of 20 mM FeCl3•6H2O solution) and the reaction mixture was incubated in a water bath at 37°C. The increase in absorbance at 593 nm was measured after 30 min. The antioxidant capacity based on the ability to reduce ferric ions of the extract was expressed as μM Trolox equivalents/ g dry weight of plant.

*ABTS free radicals scavenging assay*

The antioxidant capacity assay was carried out using the improved ABTS•+ method, as described by Re *et al.*, (29). Briefly, ABTS•+ radical cation is generated by reacting 7 mM ABTS and 2.45 mM potassium peroxodisulfate via incubation at room temperature (23°C) in the dark for 12-16 h. The ABTS•+ solution was diluted with 80% HPLC-grade ethanol to an absorbance of 0.700 ± 0.040 at 734 nm and equilibrated at 30°C. Plant extract was diluted with distilled water or 80% methanol, so that after introduction of a 30 μL aliquot of each dilution into the assay, it produced from 20% to 80% inhibition of the blank absorbance. To 3 mL of diluted ABTS•+, 30 μL of the plant extract solution was added and mixed thoroughly. The reaction mixture was allowed to stand at room temperature for 6 min and the absorbance was recorded immediately at 734 nm. Trolox standard solutions (concentrations from 0 to 32 μM) in 80% ethanol were prepared and assayed using the same conditions. Appropriate solvent blanks were run in each assay. The percent of inhibition of absorbance at 734 nm was calculated and plotted as a function of concentration of trolox for the standard reference data. The absorbance of the resulting oxidized solution was compared to that of the calibrated trolox standard. Results were expressed in terms of trolox equivalent antioxidant capacity (TEAC, μM trolox equivalents/g dry weight of plant) as described elsewhere (29).

*Lactate dehydrogenase (LDH) leakage assay*

After reperfusion, the reperfusion media was collected and centrifuged at 2000 rpm for 10 min. The supernatant was collected and the activity of leaked intracellular LDH was measured using a diagnostic kit (Merck India Ltd., Mumbai). Briefly, LDH activity was measured at 340 nm using spectrophotometer, on the basis of reduction of pyruvate to lactate in the presence of NADH, and the values expressed as Units/2 × 106 cells (30).

*Measurement of cellular viability*

Cellular viability of the cells was measured by MTT assay (31). Briefly, 10 μL of MTT was added from the stock of 5 mg/mL to 200 μL of cell suspension, followed by 4 h incubation at 37°C in CO2 incubator. Soon after, the formazon crystals formed, were pelleted out by centrifugation and dissolved in 100 μL of DMSO and the colour developed was measured spectrophotometrically at a wavelength of 570 nm and the values expressed as ^Δ^OD 570/6x104 cells.

*Measurement of cellular ATP*

After reperfusion, ATP level in the cells was measured by ATP assay kit from Calbiochem (Germany). This kit utilizes the bioluminescence for the rapid detection of the ATP levels. The enzyme luciferase catalyzes the formation of light from ATP and luciferin. The light produced was measured on a plate reader at 562 nm. Standard curve was prepared using different concentrations of ATP (0.1 ng to 100.0 ng) and the values expressed as ng/2x104 cells.

*Measurement of total lactic acid content*

Lactic acid concentration in the lysed cells and exposure media was measured by commercial diagnostic kit (Randox, (UK). In short, lactate in the presence of oxygen is converted to pyruvate and hydrogen peroxide. The reaction is catalyzed by lactate oxidase. The hydrogen peroxide in the presence of peroxidase reacts with 4-amino antipyrine and *n-*ethyl-*N*-(2-hydroxy-3-sulphopropyl)m-toluidine to yield a purple colored product that was measured spectrophotometrically at 550 nm. The concentration of lactic acid was calculated using a standard curve and expressed as μM/2x106 cells.

*Photomicrographs*

After ischemia-reperfusion photomicrographs were taken with the help of Inverted microscope (Leica DMIRB) to find out the morphological changes after ischemia.

*Statistical analysis*

For all antioxidant assays, all data are shown as mean ± SEM from three extraction replicates, each run in duplicate. Correlation and regression analysis of antioxidant activity and total phenolic content was carried out using SigmaPlot 2000 Software. For *in-vitro *methods, each experiment consisted of three separate plates from the same culture which were averaged together (n = 1). Each experiment was repeated three times using different cultures and the results expressed as mean ± SE of three different experiments. The statistical analysis was performed using one-way analysis of variance (ANOVA) followed by Student-Newman-Keuls multiple comparison test. Statistical significance was drawn at p < 0.05.

## Results and Discussion

*TPC content of F. arabica*

**Figure 1:**
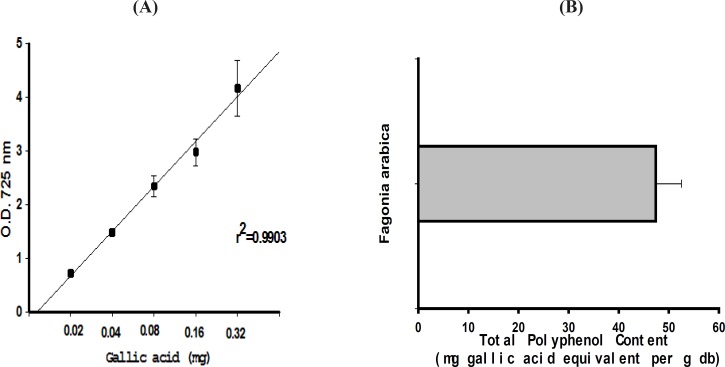
(A) Concentration-response curve at the absorbance of 725 nm for gallic acid standards with r2 = 0.9903. (B) Total polyphenol content of *F. arabica *based on the results obtained from the Folin-Ciocalteu phenol assay (mean of 3 different experiments ± SEM).

The TPC of the plant extract was determined using the Folin-Ciocalteu phenol reagent. Using Gallic acid as a standard phenolic compound, a linear calibration curve was constructed which was in the range of 20-320 μg/mL of gallic acid with r2 = 0.9903 (Figure 1A). The TPC value of *F. arabica *was found to be 47.4 ± 5.1 mg gallic acid equivalents/ g dry weight of plant (Figure 1B)

*Antioxidant activity of F. arabica*

*DPPH free radical scavenging activity*

**Figure 2:**
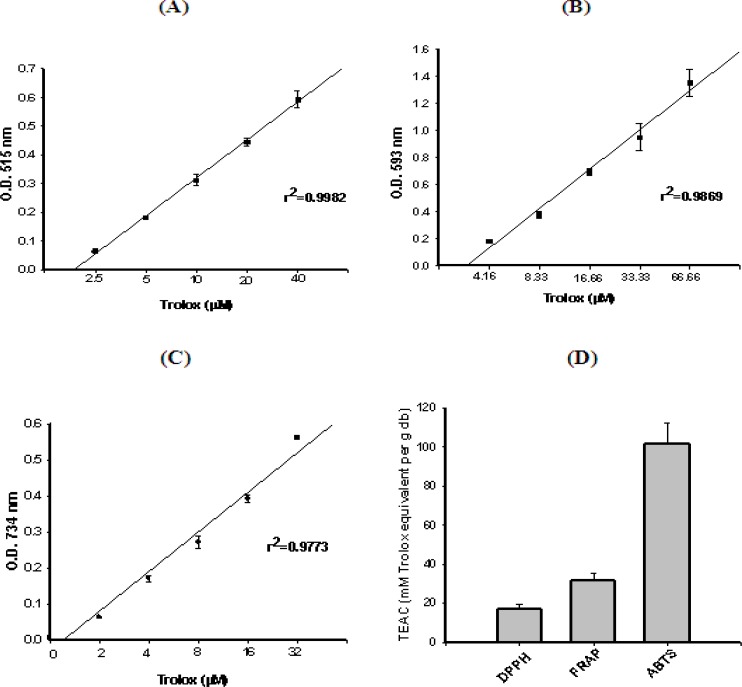
Antioxidant activity (TEAC values) of F. arabica based on its property to scavenge ABTS and DPPH free radicals and reduce ferric ions. Concentration-response curve for (A) DPPH, (B) FRAP and (C) ABTS+, as a function of standard trolox solution. (D) Antioxidant activity (TEAC values) of F. arabica (Mean of 3 different experiments ± SEM.).

The stable radical DPPH has been used widely for the determination of primary antioxidant activity i.e. the free radical scavenging activities of pure antioxidant compounds, plant and fruit extracts and food materials. The DPPH free radical scavenging activity of *F. arabica *extract is shown in Figure 2D. The concentration-response curve for DPPH (with r2 = 0.9982), as a function of five separately prepared stock solutions of trolox standards (2.5, 5.0, 10.0, 20.0, and 40.0 μM), was prepared Figure 2A.

*Ferric ion reducing activity of F. arabica*

The ability of the plant extract to reduce ferric ions was determined using the FRAP assay, which uses a single electron from an antioxidant to reduce the ferric-TPTZ (Fe(III)-TPTZ) complex into the blue ferrous-TPTZ (Fe(II)-TPTZ) complex which absorbs strongly at 593 nm. A standard curve was prepared using different concentrations of trolox (4.16, 8.33, 16.66, 33.33, and 66.66 μM), with r2 = 0.9714 (Figure. 2B). *F. arabica *showed a remarkable antioxidant activity and reduced the ferric-TPTZ to ferrous-TPTZ, significantly within the assay time (Figure 2D).

*Effect of F. arabica on ABTS free radical decolorization*

The improved ABTS•+ method, as described by Re *et al. *(29), was used to determine the antioxidant capacity of *F. arabica*. This method measures the relative antioxidant ability of the plant extract to scavenge the radical ABTS•+ in the aqueous phase. The concentration-response curve for ABTS•+ was prepared using five different concentrations (2.0, 4.0, 8.0, 16.0, and 32.0 μM) of trolox which was prepared with r2 = 0.9733 (Figure. 2C). Figure. 2D indicates that *F. arabica *showed appreciable antioxidant activity, which was found to be around 101.76 mM TEAC (μM trolox equivalents/ g dry weight of plant).

*Effect of F. arabica on cell viability*

**Figure 3:**
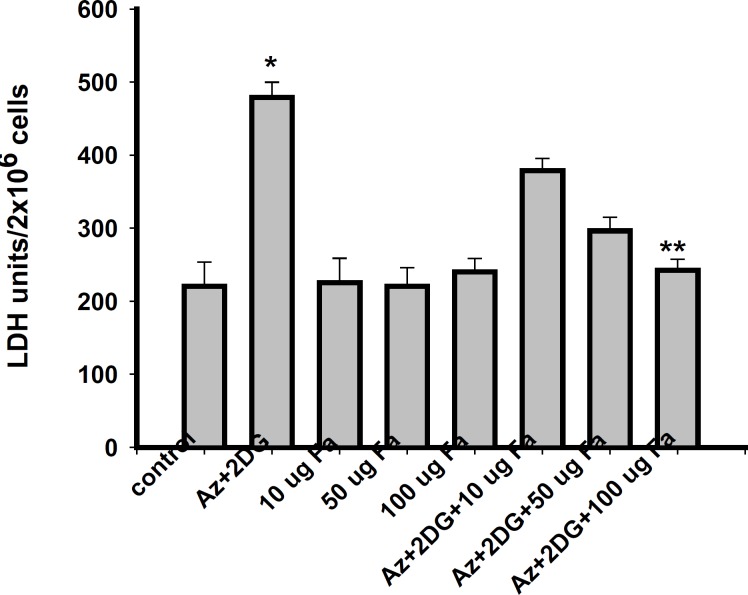
Mitochondrial integrity of PC12 cells as measured by MTT test. Values are mean ± S.E. of three different experiments. *Significantly different from normal control, **significantly different from ischemic control, at p < 0.05 (ANOVA–Student–Newman–Keuls).

**Figure 4:**
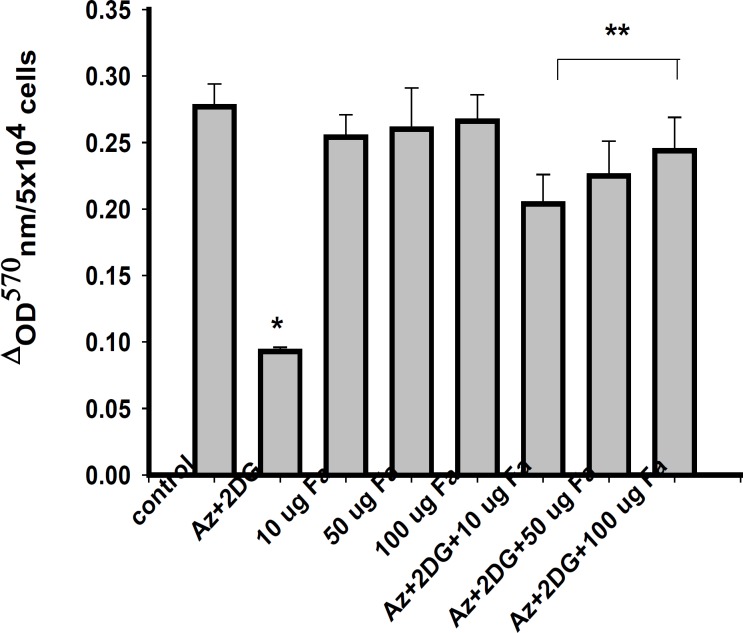
Leakage of lactate dehydrogenase from PC12 cells after ischemia/ reperfusion. Values are mean ± S.E. of three different experiments. *Significantly different from normal control, **significantly different from ischemic control, at p < 0.05 ; ANOVA–Student–Newman–Keuls

To determine the effect of *F. arabica *on neuronal cell death, cell membrane integrity (LDH leakage) and mitochondrial function (MTT assay) were measured. Viability of cells significantly reduced after ischemia/reperfusion period. *F. arabica *at any given concentration did not show any deleterious effects on the cell viability, as well as on LDH leakage when compared to control. The cytotoxicity induced by ischemia was reduced to a significant extent when cells were treated with 100 μg *F. arabica *(Figure. 3). Similarly, the membrane integrity of the cells was found to be maintained when cells were treated with *F. arabica *at the concentration of 100 μg (Figure 4).

*Effect of F. arabica on cellular ATP content*

**Figure 5:**
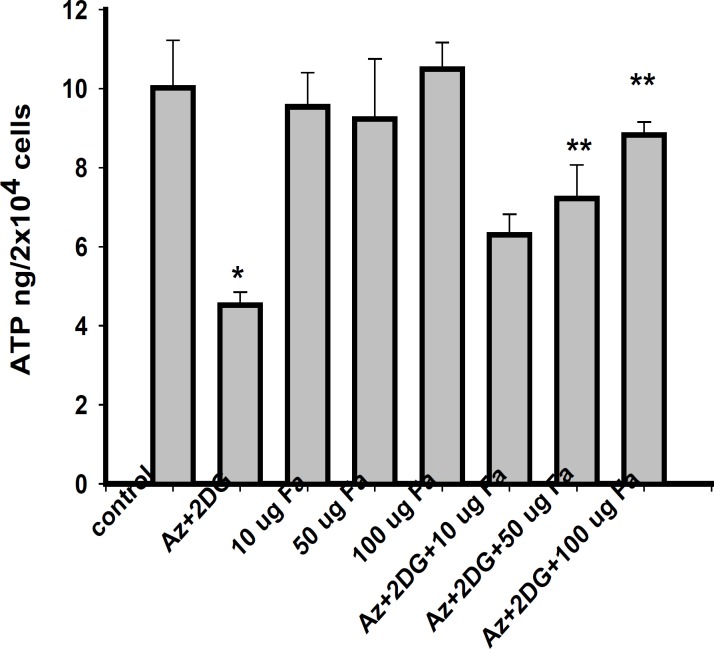
ATP levels in PC12 cells after 2 h of ischemia and 24 h of reperfusion. Values are mean ± S.E. of three different experiments. *Significantly different from normal control, **significantly different from ischemic control, at p < 0.05 (ANOVA–Student–Newman–Keuls

*F. arabica *alone did not alter the ATP levels of the cells as compared to control. The 100 μg *F. arabica *treatment restored the ATP levels in ischemic cells, but failed to do so at 10 and 50 μg concentrations (Figure 5). 

*Effect of F. arabica on cellular lactic acid content*

**Figure 6:**
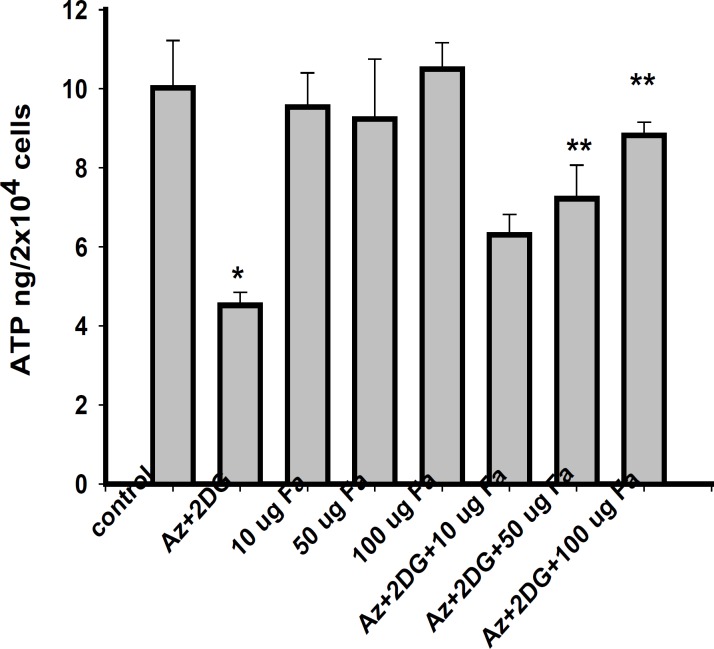
Total lactic acid content of PC12 cells after 2 h of ischemia and 24 h of reperfusion. Values are mean ± SE of three different experiments. *Significantly different from normal control, **significantly different from ischemic control, at p < 0.05 (ANOVA–Student–Newman–Keuls).

Total lactic acid content of the cells was found to increase significantly when the cells were treated with azide alone and in ischemia/reperfusion, but not in the cells treated only with 2-DG. 100 μg of *F. arabica *was found to be effective in limiting the lactic acid production, while 10 and 50 μg of *F. arabica *was found to be ineffective (Figure 6).

**Figure 7:**
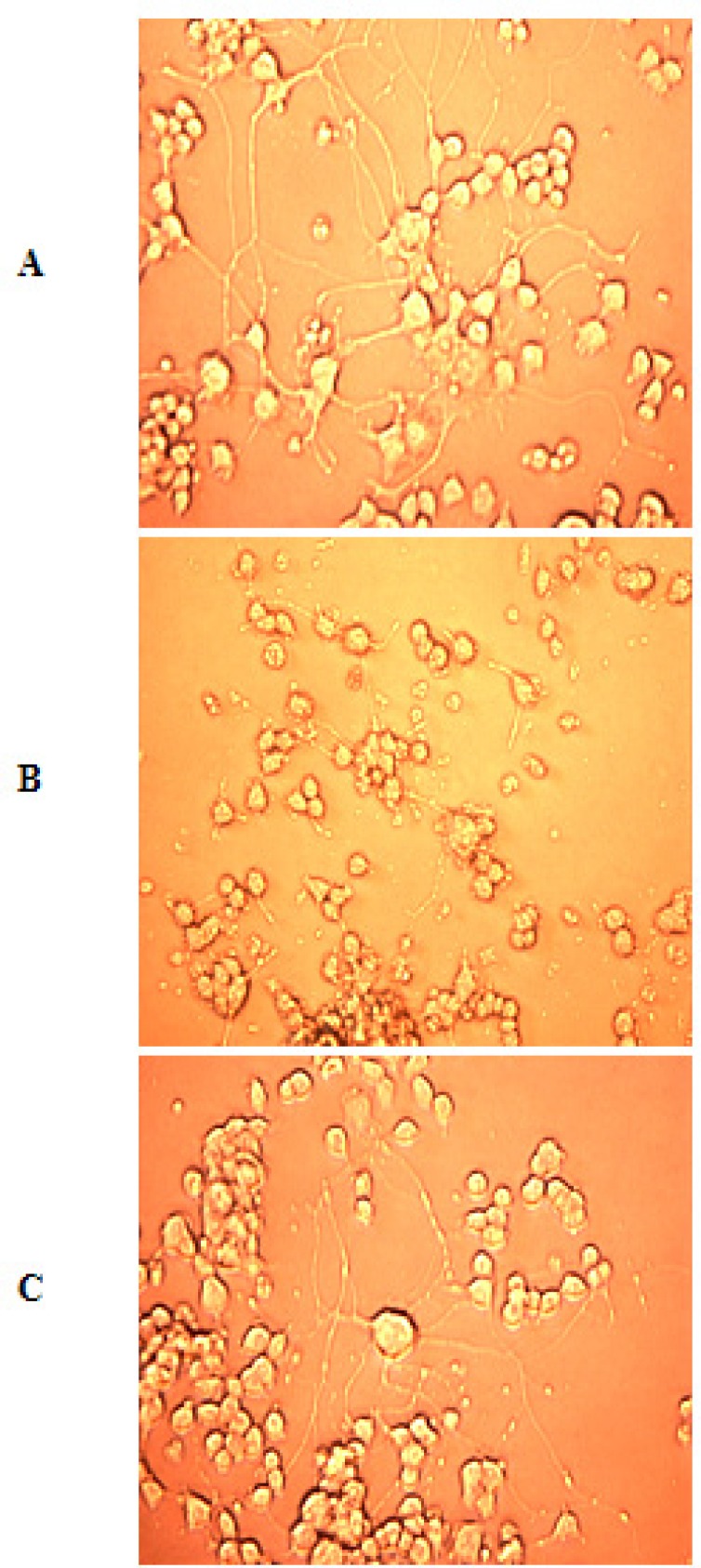
A = Normal differentiated PC12 cells showing normal morphology and neurite outgrowth. B = Ischemic cells showing neurite degeneration, disturbed cellular membrane and cell to cell contact. C = Protection of PC12 cells in the presence of *F. arabica*. (Images at 20X).

In this study, we have investigated the effect of *F. arabica *on the cytotoxicity, cellular energy status and ROS production in the chemical ischemia/reperfusion induced PC12 cells. To induce hypoxia/hypoglycemia, sodium azide (5.0 mM) and 2-deoxyglucose (2.0 mM) were used (24). Both sodium azide and 2-DG induced cell death in a concentration-dependent manner (data not shown). The induction of chemical ischemia followed by post-incubation (reperfusion) in normal medium for 24 h inflicted lethal damage to the cells, accompanied by decrease in cellular ATP content. A significant increase in glutamate levels in the supernatant of the incubation buffer has been reported after the induction of chemical ischemia in rat cortical cultures (32). Moreover, the chemical ischemia induced neurotoxicity was prevented by NMDA receptor antagonists MK-801 and D-2-amino-5-phosphonovaleric acid (APV) indicating the suitability of chemical ischemia in screening of neuroprotective agents (32, 33).

There is currently an upsurge of interest in phytochemicals as potential new sources of natural antioxidants. The goal is to use them in foods and pharmaceutical preparations to replace synthetic antioxidants (34, 35, 26). Most antioxidants isolated from higher plants are polyphenols. In vascular plants, more than 4000 phenolic and polyphenolic compounds have been identified (*e.g. *phenolic acids, tannins, coumarins, anthraquinones, flavonoids (36, 37). A wide range of low and high molecular weight plant polyphenols with antioxidant properties has been studied (38). The antioxidant activity of phenolic compounds is mainly due to their redox properties, which allow them to act as reducing agents, hydrogen donators, and singlet oxygen quenchers. In addition, they have metal-chelating potential (39). Moreover, phenolic compounds show different biological activities as antibacterial, anticarcinogenic, anti-inflammatory, anti-viral, anti-allergic, estrogenic, and immune-stimulating agents (40). *F. arabica *shows a considerable amount of total phenolic content and thus in turn shows considerable antioxidant activities.

*F. arabica *is an Ayurvedic medicine popular in a number of countries particularly in the sub-Saharan countries and Indian subcontinent, for its anti-inflammatory activity and considerable analgesic and antipyretic effects (14, 40, 41). Many triterpenoids and saponins and their chemical structures has been reported from different species of genus Fagonia (20, 21). Most of the flavonol glycosides have been isolated from various Egyptian Fagonia species and their phylogenetic affinities have also been investigated (42-48). Other constituents, such as docosyl docosanoate from hexane extract and water-soluble proteins from aqueous extract of air-dried F. cretica plants have been isolated (49, 50) furthermore, nahagenin (51) hederagnin, ursolic acid and pinitol from other Fagonia species have also been separated and characterized (52). The antimicrobial activity of its flavonoid compounds has been explored previously, (53) while the nutritive values of it and of other species growing wild in the Rajasthan region of India, have also been evaluated (54). Very recently the thrombolytic activity of *F. arabica *has been demonstrated (55).

Antioxidant activity of *F. arabica *was evaluated by various parameters. Considerable amount of total polyphenols was measured in *F. arabica *indicating it’s prospective as a potent antioxidant. The antioxidant activity of herbal extract was evaluated with the help of ABTS, DPPH and FRAP assay. *F. arabica *reduces the ABTS•+ radicals significantly, indicating its potential antioxidant activity. The model of scavenging the stable DPPH radical is a widely used method for evaluating antioxidant activities in a relatively short time compared with other methods. The effect of antioxidants on DPPH radical scavenging was thought to be due to their hydrogen donating ability (56). The trend for ferric ions reducing activity (FRAP) of *F. arabica *was almost double than that of DPPH. The probable reason for the lower DPPH values of the extract could be due the presence of compounds not reactive towards DPPH. Antioxidant compounds such as polyphenols may be more efficient reducing agents for ferric iron but some may not scavenge DPPH free radicals as efficiently due to steric hindrance.

Hypoxia stimulates anaerobic glycolysis and results in enhanced lactate production, resulting in lactic acidosis. A significant rise in the lactic acid concentration was observed after 2 h of ischemia in PC12 cells. Removal of lactic acid from the extracellular space is largely due to an uptake mechanism, which is an ATP dependent process. Ischemia causes ATP depletion within 5 min. therefore the lactic acid uptake mechanism is blocked and lactic acid accumulates (57). *F. arabica *treatment shows a remarkable increase in ATP levels and probably due to which lactic acid levels returns to normalcy.

 Several studies are going on throughout the world to identify antioxidant compounds that are pharmacologically potent with low profile of side effects. Ayurveda, the oldest medical system in the world, provides lots of lead to find active and therapeutically useful compounds from plants. *F. arabica *by virtue of its antioxidant potential reduces oxidative stress generated due to ischemia-reperfusion and help the cells to maintain the cellular ATP and lactic acid levels, thus ultimately prevents cell death due to ischemia/reperfusion. Additional work in this area is currently underway to find out the active components of *F arabica *and their mode of action.
